# Correction: Sub-millimetre resolution laminar fMRI using Arterial Spin Labelling in humans at 7 T

**DOI:** 10.1371/journal.pone.0251774

**Published:** 2021-05-12

**Authors:** The PLOS ONE Staff

A duplicate of S1 Fig was incorrectly published as S10 Fig. Please view the correct S10 Fig below. The publisher apologizes for the error introduced during the typesetting process.

## Supporting information

S10 Fig (left-to-right) Single-subject and average laminar profiles of the positive response during stimulation for the BOLD signal, absolute and relative perfusion change with cortical depth along the X-axis. (TIF)
