# Supplementary figures and images for: Sub-millimetre resolution laminar fMRI using Arterial Spin Labelling in humans at 7 T
Source: PLoS One. 2021 Apr 26;16(4):e0250504. doi: 10.1371/journal.pone.0250504 (PMC8075193; doi:10.1371/journal.pone.0250504)

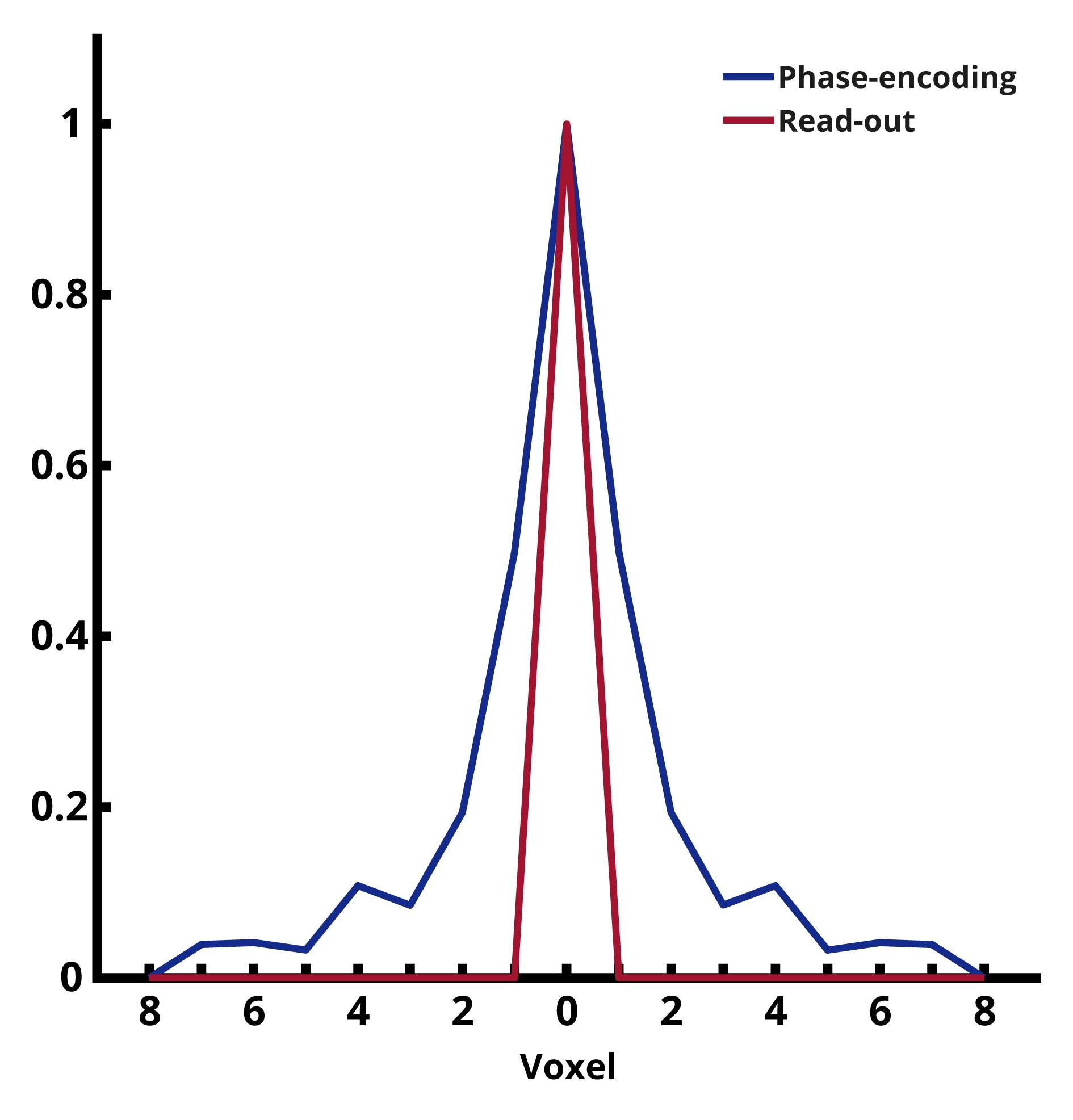

Supplement: S1 Fig — Partial-Fourier [117] was employed along the principal phase encoding direction. Please note, this simulation used zero-filling with partial-Fourier representing the default image reconstruction scenario. However, in the present study, a POCS reconstruction [59] with 8 iterations was carried out which minimises partial-Fourier blurring, and consequently improves the PSF [57]. (TIF) [file pone.0250504.s001.tif]

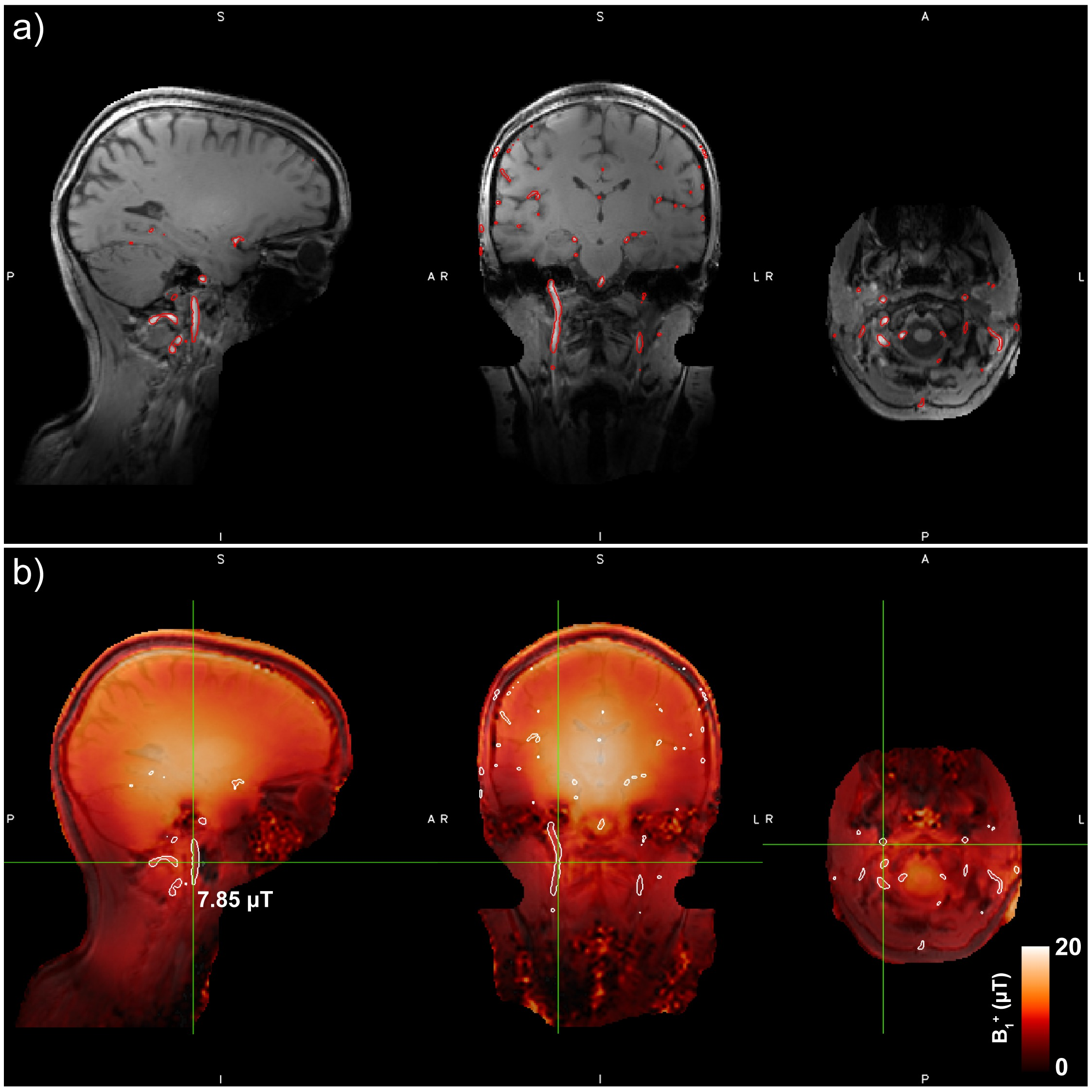

Supplement: S2 Fig — (a) The red outlines are the segmented arteries using a separate time-of-flight (ToF) scan and segmented using braincharter (https://github.com/braincharter/vasculature) [125]. (b) The B1+ map is overlaid on the second inversion image of the MP2RAGE with the arteries overlaid as white outline. At the point of the cross-hair (internal carotid artery), the B1+ value is measured to be 7.85 μT in this participant, which is in line with the 95% inversion efficiency threshold for our TR-FOCI pulse. Therefore, the arterial water spins in the feeding vessels are adequately labelled in the present study. (TIF) [file pone.0250504.s002.tif]

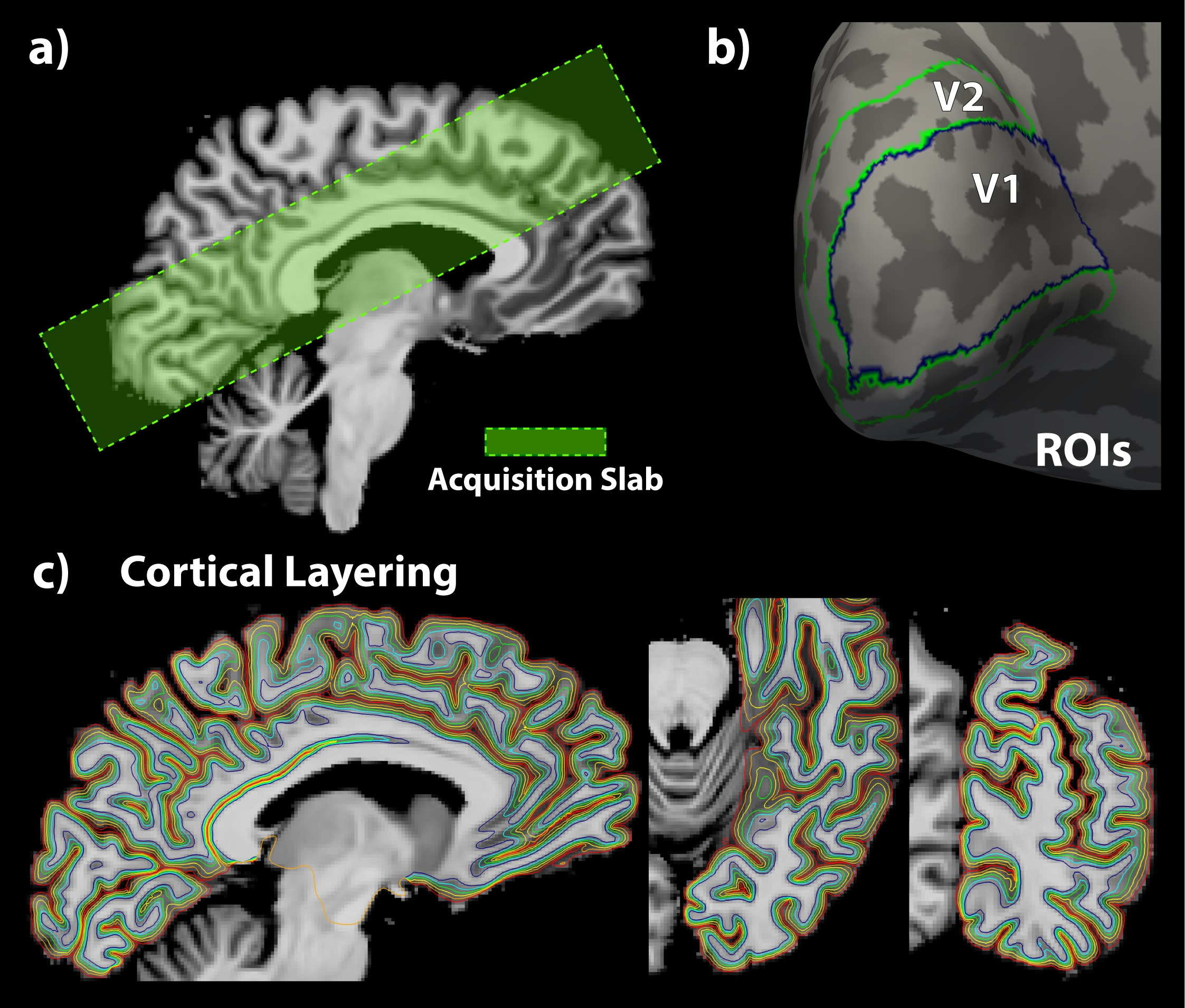

Supplement: S3 Fig — (TIF) [file pone.0250504.s003.tif]

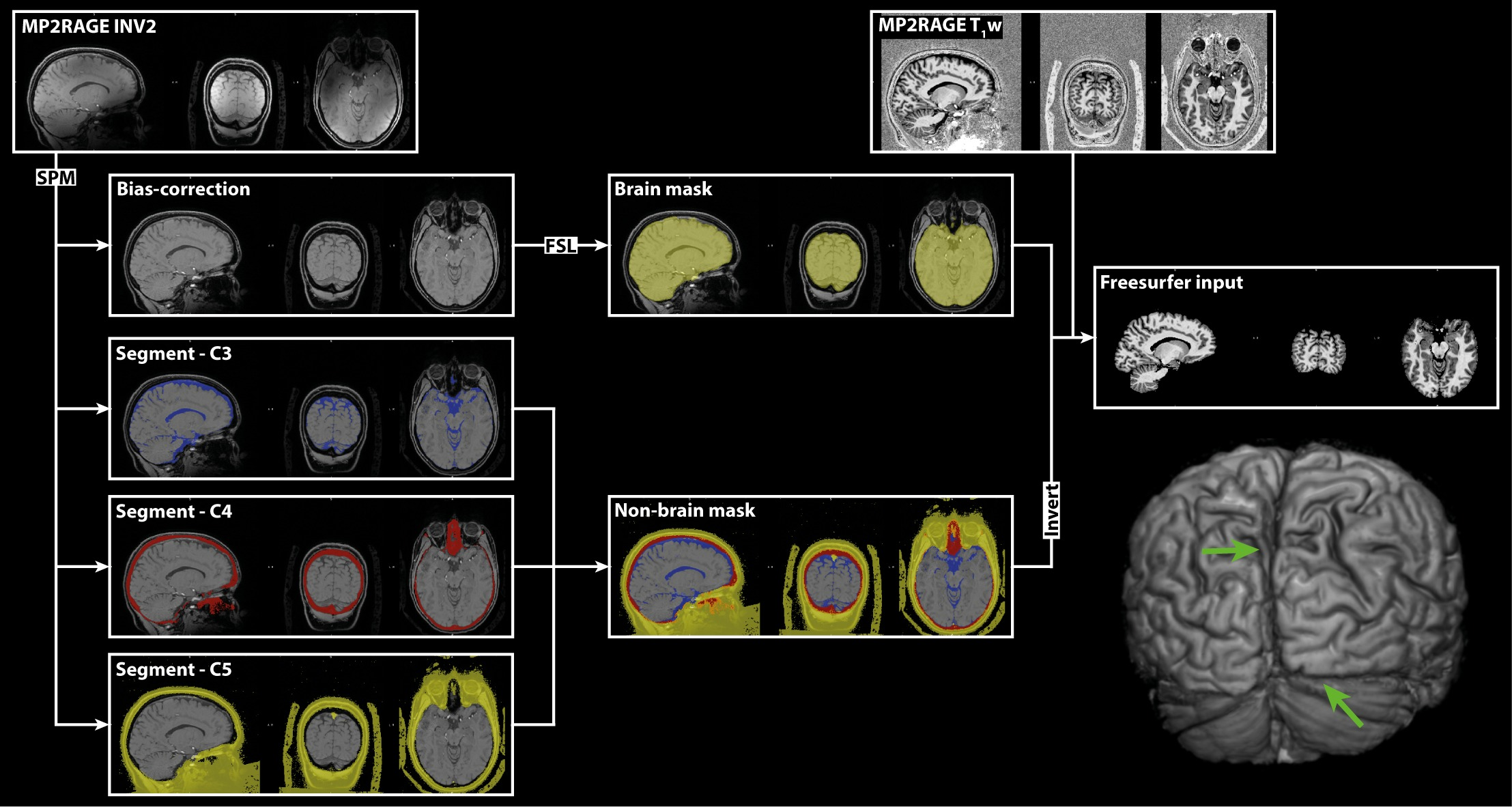

Supplement: S4 Fig — Tissue classes C3-C5 were thresholded on a subject-by-subject basis to include as much of the sagittal and transverse sinuses as possible. In most of our subjects, this procedure did not require any manual correction of the combined mask. ITK-SNAP v3.6 [72] was used to make any manual corrections when required. The Freesurfer T1-weighted input is presented as a 3D render showing an intact GM surface at the occipital lobe with the green arrows indicating locations of the now automatically stripped sagittal and transverse sinuses. (TIF) [file pone.0250504.s004.tif]

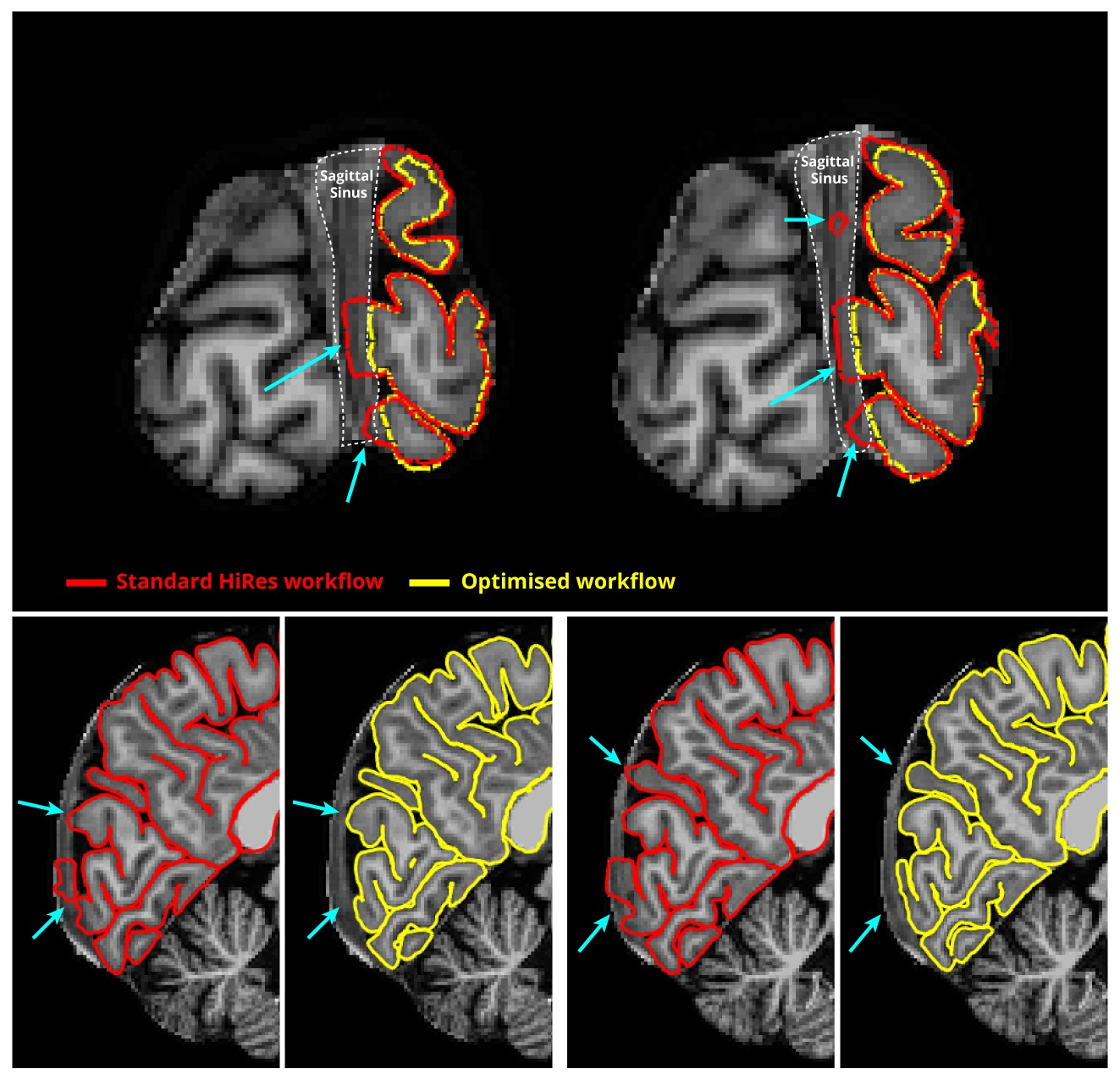

Supplement: S5 Fig — (top) Coronal view of the pial surface overlaid on a standard skull-stripped T1-weighted MP2RAGE image. Shown in red is determined from the standard HiRes workflow and in yellow is determined from the optimised workflow shown in S4 Fig. White dotted lines indicate sagittal sinus and cyan arrows emphasise the erroneous placement of the pial surface in the standard workflow. (bottom) Sagittal views of the pial surface from the standard and optimised workflows to further illustrate their differences in outcome indicated by the cyan arrows. (TIF) [file pone.0250504.s005.tif]

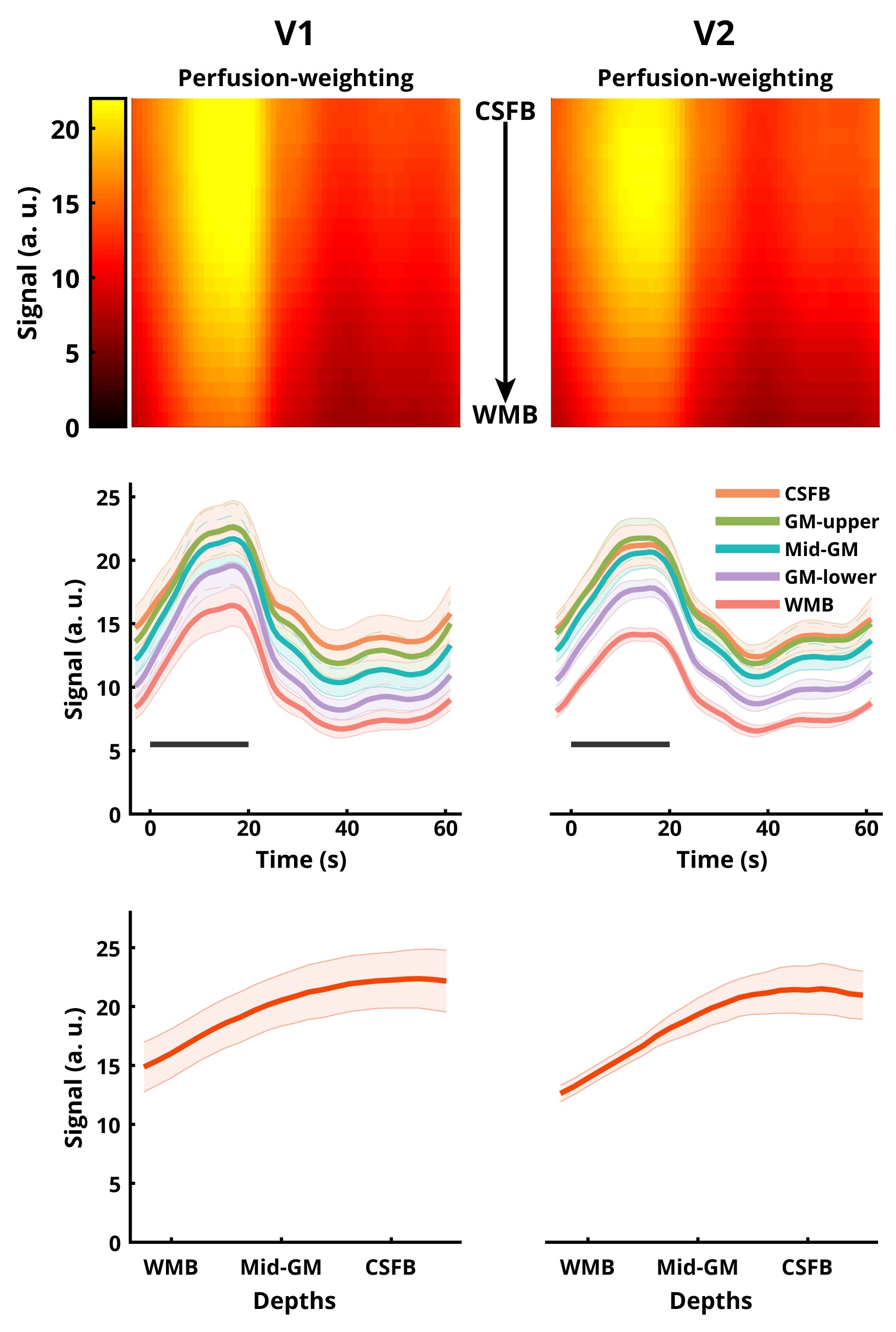

Supplement: S6 Fig — (top row) Heatmap representations of the group-average perfusion-weighted signal with cortical depth along Y-axis and Time along the X-axis. (middle row) Five out of the twenty-three total laminar time-courses and (bottom row) laminar profiles of the positive response for the perfusion-weighted signal. All error-bars indicate SEM. The grey bar in the middle row indicates the stimulus duration. (TIF) [file pone.0250504.s006.tif]

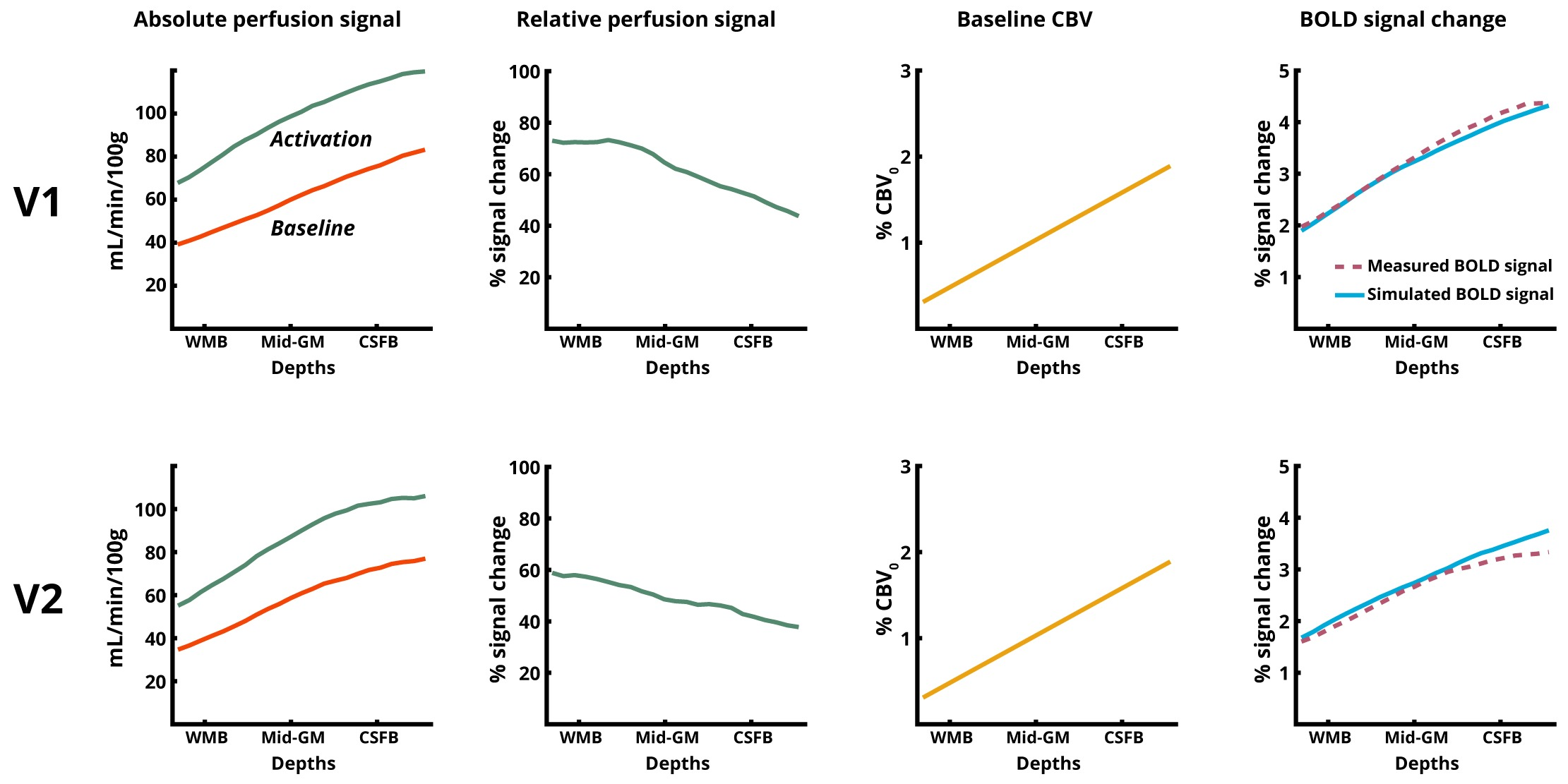

Supplement: S7 Fig — (TIF) [file pone.0250504.s007.tif]

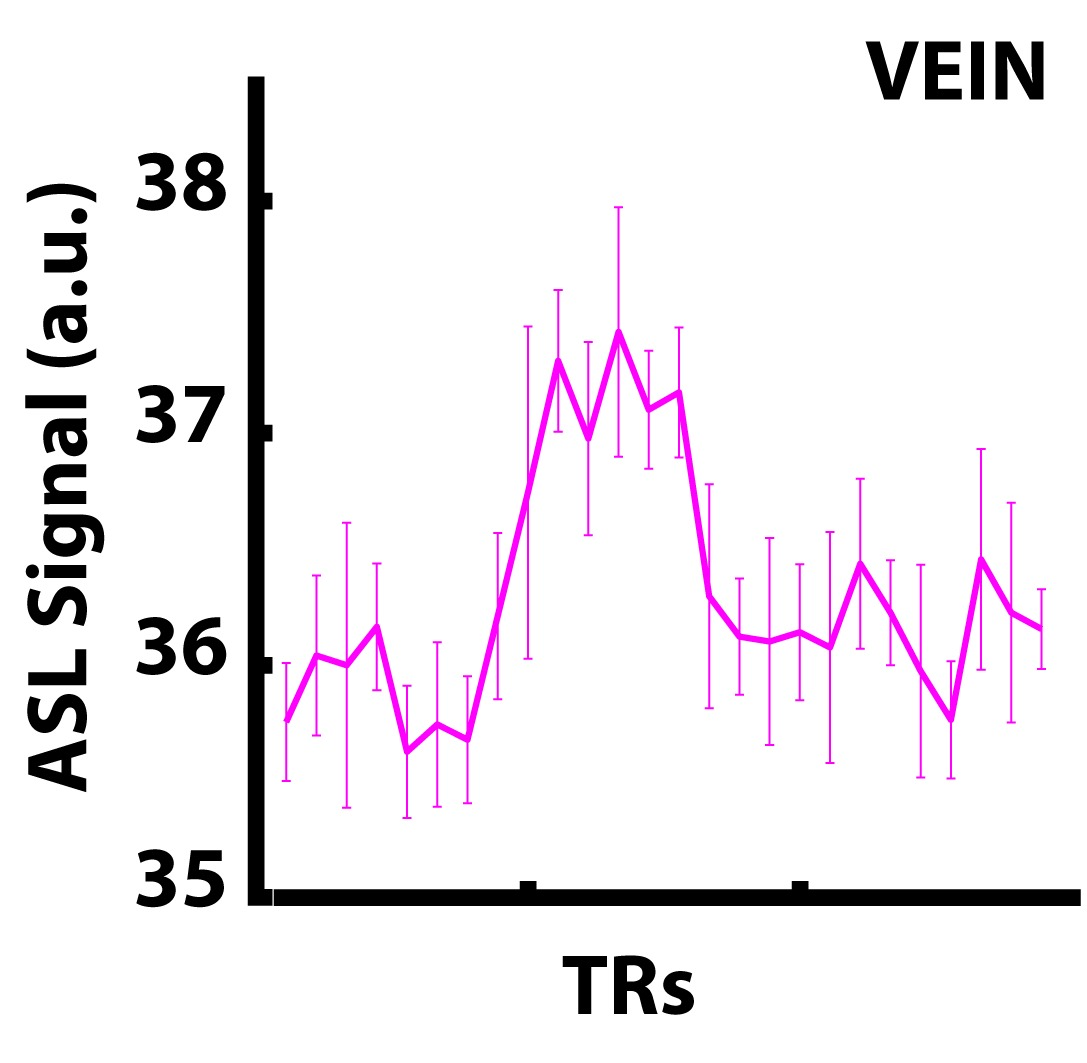

Supplement: S8 Fig — Error bars indicate SEM across trials. Please note the near absence of the characteristic ASL zig-zag signal modulation and the small scale of the Y-axis (in MRI signal units). (TIF) [file pone.0250504.s008.tif]

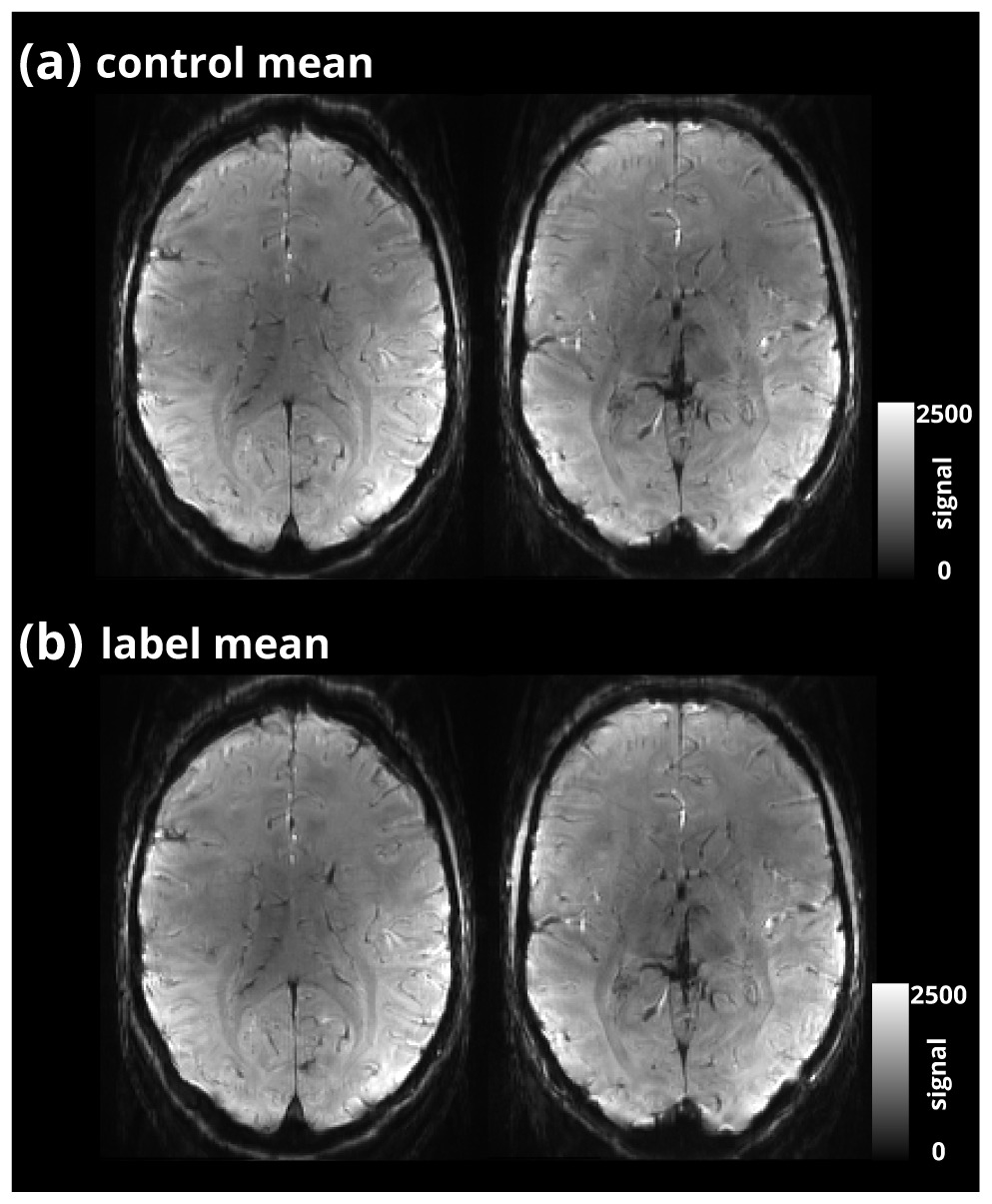

Supplement: S9 Fig — (TIF) [file pone.0250504.s009.tif]
