# Supplementary figures and images for: Correction: Sub-millimetre resolution laminar fMRI using Arterial Spin Labelling in humans at 7 T
Source: PLoS One. 2021 May 12;16(5):e0251774. doi: 10.1371/journal.pone.0251774 (PMC8115793; doi:10.1371/journal.pone.0251774)

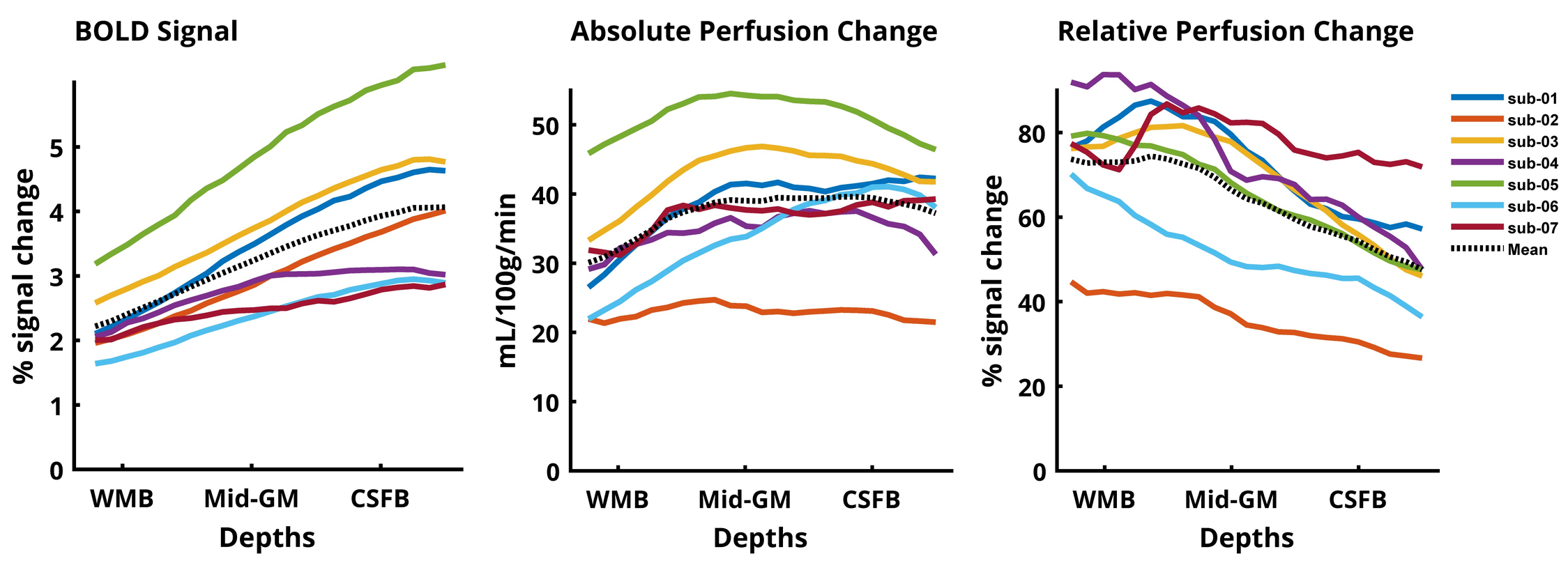

Supplement: S10 Fig — (TIF) [file pone.0251774.s001.tif]
